# The Next Pandemic: On the Front Lines Against Humankind’s Gravest Dangers

**DOI:** 10.3201/eid2312.171137

**Published:** 2017-12

**Authors:** William P. Hanage

**Affiliations:** Harvard T.H. Chan School of Public Health, Boston, Massachusetts, USA

**Keywords:** field epidemiology, outbreaks, policy, pandemics

Stalin notoriously remarked that one death was a tragedy, while a million were a statistic. An impressive feature of this book is that, while recounting a great many deaths, it maintains a human and humane perspective. Dr. Ali S. Khan is a former director of the Office of Public Health Preparedness and Response at the Centers for Disease Control and Prevention and has been close to the epicenter of almost all the potential pandemic threats of the last few decades. With William Patrick, an accomplished writer and editor in his own right, Khan has produced an accessible, fascinating book for the general reader that shares his experiences in the service of his country and the world (Figure). 

**Figure:**
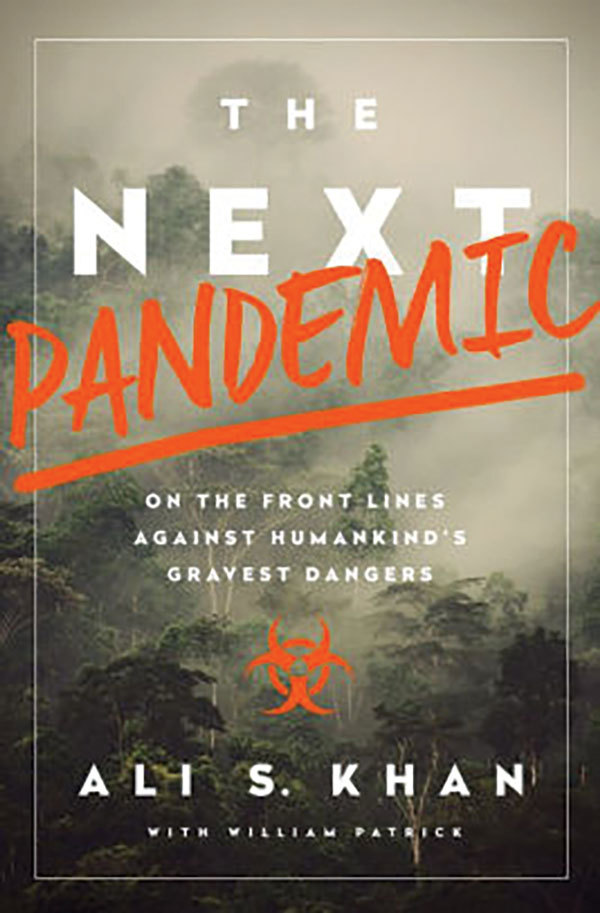
The Next Pandemic: On the Front Lines Against Humankind’s Gravest Dangers

The book describes so many outbreaks that its approach is inevitably a little scattershot, but 2 subjects stand out: the investigation of the anthrax mailings to Congress is covered as well as I have seen anywhere, and the sections dealing with the nightmare of Ebola in West Africa should be required reading for anyone looking to improve our response to infectious disease emergencies. As the authors note, maintaining high standards of data hygiene is difficult anywhere, but particularly so if we lack the will to prioritize public health even when resources are scarce.

Throughout, there is a subtle but steady bass line of anguish over the difficulty of translating the science we know into effective action. Khan argues that to keep our priorities straight we need to recognize how the unscrupulous, from black marketeers to politicians, exploit outbreaks as opportunities for advancement; addressing that issue is an essential part of fighting the disease. In the words of Albert Camus, “It is up to us not to join forces with the pestilences.” We might not, but how can we stop others from doing so? Khan suggests the creation of a United Nations Undersecretary for Health Security, which sounds sensible, but it is not clear what policies that person might enact, and even less so how they would be enforced.

It is the personal stories here that are the most affecting, in the understanding that Khan extends to an Ebola-infected nurse who denies that she knows how she acquired the disease, or the prejudice faced by Khan himself as a Muslim American in the years following 9/11. These diseases infect humans, kill humans, and are battled by humans, with all the complicated consequences that entails.

There are a few missteps. The persistent nagging use of clichés like “disease detectives” can sound patronizing. An assertion that viruses are “collectively intelligent” will surely raise eyebrows, and the title is a little misleading, in that there is less focus than expected on genuine catastrophic pandemic threats. However, as another confessed “infectious disease nerd” I found it fascinating. It will be enjoyed by working epidemiologists; it should be a point of reference for policymakers; and I suspect it will inspire many future officers in the Epidemic Intelligence Service.

